# Intracoronary application of nicorandil regulates the inflammatory response induced by percutaneous coronary intervention

**DOI:** 10.1111/jcmm.15169

**Published:** 2020-03-16

**Authors:** Keqing Hu, Xiaoqi Wang, Hongyan Hu, Zhongyang Xu, Jiaxing Zhang, Guipeng An, Guohai Su

**Affiliations:** ^1^ Cardiovascular Department Jinan Central Hospital Affiliated to Shandong University Jinan Shandong China; ^2^ The Key Laboratory of Cardiovascular Remodeling and Function Research The State and Shandong Province Joint Key Laboratory of Translational Cardiovascular Medicine Chinese Ministry of Education Chinese National Health Commission and Chinese Academy of Medical Sciences Shandong University Qilu Hospital Jinan Shandong China

**Keywords:** inflammation, myocardial no‐reflow, nicorandil, percutaneous coronary intervention, proteomic analysis

## Abstract

Intracoronary application of nicorandil can effectively reduce the myocardial no‐reflow (MNR) after percutaneous coronary intervention (PCI). We sought to investigate the mechanisms of nicorandil in preventing MNR, besides that of dilating the coronary microvasculature. A total of 60 patients undergoing PCI were enrolled and randomly divided into a nicorandil group and a control group. Before PCI, 2 mg of nicorandil or an equal volume of normal saline was injected into the coronary artery. Blood samples were collected before, 24 hours and 1 week after PCI and inflammatory cytokines were tested. In the control group, the expression of pro‐inflammatory cytokines was significantly increased, while the anti‐inflammatory cytokines were decreased 24 hours after PCI. In contrast, these changes were reversed in the nicorandil group, indicating that nicorandil regulated the inflammatory response induced by PCI. Then, proteomic analysis was performed to further elucidate the potential mechanisms. A total of 53 differentially expressed proteins (DEPs) were found 24 hours after PCI in the control group, and the changes of these relevant genes were reversed in the nicorandil group. These DEPs were significantly enriched in the inflammatory pathways. In conclusion, the intracoronary application of nicorandil before PCI can regulate the inflammatory responses induced by PCI, which might be an important mechanism of nicorandil in preventing MNR.

## 1 INTRODUCTION

Percutaneous coronary intervention (PCI) has become an important treatment for acute myocardial infarction (AMI). Compared with traditional drug therapy, PCI can restore myocardial perfusion more effectively, reduce the area of myocardial ischaemia or infarction and improve the clinical prognosis. However, despite continuing advances and improvements in PCI technology, some patients still fail to benefit from PCI treatment, showing either suboptimal perfusion after PCI, or no perfusion; that is, no‐reflow of arterial blood in the myocardium (myocardial no‐reflow, MNR).^1^, ^2^ MNR is a strong predictor of poor short‐ and long‐term prognosis and major cardiovascular adverse events in patients with acute coronary syndrome (ACS) following PCI, which often leads to morbidities such as cardiac dysfunction, progressive myocardial damage and arrhythmia.^3^, ^4^

Previous studies have shown that there are several mechanisms contributing to MNR, such as coronary microvascular structure or function disorders, local metabolic changes such as those caused by oxygen free radicals, inflammation, microcirculation mechanical embolization and coronary spasm. At present, it is considered that the MNR phenomenon is a manifestation of microcirculation disturbances following myocardial ischaemia/reperfusion injury.^1^ Finding an effective way to prevent the occurrence of MNR following PCI is an urgent problem currently awaiting a solution. Many interventions to improve myocardial perfusion have been trialled, including mechanical methods such as distal devices and thrombus aspiration devices, and pharmacological methods, such as platelet glycoprotein GPIIb/IIIa antagonists and vasodilators.^5^, ^6^

Nicorandil is an ATP‐sensitive potassium channel (K_ATP_) opener. It has the properties of both nitrate‐like agent and K_ATP_ opener and acts to increase the concentration of cyclic guanylate (cGMP) in vascular smooth muscle cells and dilate blood vessels.^7^ In recent years, many researchers have tried to use intravenous or intracoronary nicorandil to improve coronary microcirculation and myocardial perfusion during PCI and reduce myocardial ischaemia‐reperfusion injury. Nicorandil administration before PCI in patients with AMI can significantly improve coronary blood flow, reduce the occurrence of ventricular arrhythmias and improve left ventricular function.^8^, ^9^, ^10^ Although there is a lack of large‐scale, multi‐centre, double‐blind controlled clinical data, most clinical studies have shown that, compared with nitroglycerin, it can better correct coronary no‐reflow, increase myocardial perfusion and improve cardiac function.

Most of the past studies of nicorandil and coronary blood flow have taken the improvement of coronary blood flow as the observation index, but the specific mechanism of the effect of nicorandil on coronary microcirculation disturbance remains unclear. Does nicorandil improve myocardial perfusion in other ways, in addition to dilating coronary vessels? In the present study, we sought to explore the potential mechanism of nicorandil in reducing the incidence of MNR in patients with PCI.

## 2 MATERIALS AND METHODS

### 2.1 Study population

This study population was derived from a consecutive series of patients with ACS, including acute ST segment elevation myocardial infarction (STEMI), non‐ST segment elevation myocardial infarction (NSTEMI) and unstable angina pectoris (UA), who underwent PCI. Exclusion criteria included: (a) hepatic impairment: that is, alanine aminotransferase (ALT) and aspartate aminotransferase (AST) three times or more above the upper limit of normal values; (b) renal insufficiency: that is, creatinine > 2.5 mg/dL (221 µmol/L); (c) patients with malignant tumours; (d) patients with primary or secondary thrombocytopenia or dysfunction; (e) patients with moderate to severe anaemia, that is, Hgb < 90 g/L; (f) a previous medical history of PCI or coronary artery bypass graft (CABG); (g) acute heart failure; (h) those allergic to nicorandil.

The study protocol was approved by the local ethics committee, and written informed consent was obtained from all participants. All participants were randomly divided on a 1:1 basis into a nicorandil group and a control group. Patients in the nicorandil group were given 2 mg of nicorandil injected into the coronary artery at 2 mm beyond the occlusion, with balloon pre‐dilation prior to PCI. Those patients in the control group received an equal volume of normal saline injected in the coronary artery prior to PCI. All patients were given standard medications, including: dual antiplatelet drugs (aspirin 300 mg once and 100 mg daily, ticagrelor 180 mg once and 90 mg twice daily), statins (atorvastatin 40 mg daily), β‐receptor blockers (unless contraindicated) and angiotensin‐converting enzyme inhibitor (ACEI) (unless contraindicated).

### 2.2 Blood sample collection

Venous blood samples were collected from all participants before undergoing PCI, 24 hours after PCI and 7 days after PCI. Whole blood samples were collected from them in heparin‐containing vacutainer tubes, and then centrifuged at 1,000 *g* for 15 minutes at 25°C to obtain the fresh serum. The serum was immediately aliquoted into sterile centrifuge tubes and frozen at −80°C for analysis.

### 2.3 Cytokines detection

Series cytokines were tested for, including tumour necrosis factor‐alpha (TNF‐α), interleukin (IL)‐1β, IL‐1 receptor A (IL‐1RA), IL‐6, IL‐10, IL‐19, IL‐33, IL‐18, vascular cell adhesion molecule 1 (VACM‐1), intercellular adhesion molecule‐1 (ICAM‐1), monocyte chemoattractant protein‐1 (MCP‐1), chemokine ligand 1 (CXCL‐1) and CXCL‐2. Cytokine concentrations of plasma were determined by enzyme‐linked immunosorbent assay (ELISA) kits (R&D Systems) according to the manufacturer's instructions. All cytokines were standardized by inclusion of a titration of the appropriate purified recombinant cytokine of known concentration. The absorbance of the samples was determined on an MRK Microplate Reader with 450 nm as the wavelength.

### 2.4 Proteomic analysis

The serum total proteins of the patients in both the nicorandil and control groups were extracted before PCI, 24 hours after PCI and 7 days after PCI, for the purposes of proteomic analysis. A total of three biological replicates for each of the groups at each time‐point were used for proteomic analysis. Gene Ontology (GO) and KEGG (Kyoto Encyclopedia of Genes and Genomes) were used to analyse the protein family and pathway.

### 2.5 Statistical analysis

Data were analysed with SPSS 16.0 (SPSS Inc). Quantitative values were expressed as mean ± standard error of the mean (SEM) and analysed by unpaired t test or one‐way ANOVA as appropriate. A level of *P* < .05 was considered significant.

## 3 RESULTS

### 3.1 Clinical characteristics

A total of 60 patients with ACS were enrolled in the study and divided into two groups randomly: a nicorandil group and a control group (n = 30 for each group). The nicorandil group included 12 patients with STEMI, nine with NSTEMI and nine with UA. The control group included 11 with STEMI, 11 with NSTEMI and eight with UA. The clinical characteristics and CAD risk factors for all participants were recorded. As shown in Tables 1 and 2, the two groups did not differ in age, sex, obesity, smoking, history of diabetes, hypertension, blood pressure, lipid levels or fasting blood glucose.

**Table 1:** Clinical characteristics of the study population

Characteristics	Nicorandil group (n = 30)	Control group (n = 30)
Male (%)	19 (63.33%)	22 (73.33%)
Age (years)	59.97 ± 1.32	60.43 ± 1.26
STEMI (n, %)	12 (40%)	11 (36.67%)
NSTEMI (n, %)	9 (30%)	11 (36.67%)
UAP (n, %)	9 (30%)	8 (26.67%)
BMI (kg/m^2^)	24.77 ± 0.67	25.22 ± 0.61
Smoking (%)	11 (36.67%)	14 (46.67%)
Diabetes history	12 (40%)	10 (33.33%)
Hypertension	19 (63.3%)	21 (70%)
Systolic BP (mmHg)	131.17 ± 3.90	130.03 ± 2.96
Diastolic BP (mmHg)	75.03 ± 2.48	79.63 ± 1.72
Heart rate (bpm)	70.00 ± 1.72	72.07 ± 2.66

Note

Data are expressed as mean ± SEM or number (%) of participants. There were no significant differences in the clinical characteristics between the patients in the control group and the nicorandil group.

Abbreviations: BMI, body mass index; BP, blood pressure; NSTEMI, non‐ST segment elevation myocardial infarction; STEMI, acute ST segment elevation myocardial infarction; UAP, unstable angina pectoris.

**Table 2:** Haematology test indicators of participants in the two groups

Parameters	Nicorandil group (n = 30)	Control group (n = 30)
WBC (10^9^/L)	6.39 ± 0.30	6.67 ± 0.31
Haemoglobin (g/L)	134.10 ± 2.62	137.73 ± 3.48
Platelet (10^9^/L)	234.20 ± 9.28	229.87 ± 10.65
ALT (U/L)	19 (63.33%)	22 (73.33%)
AST (U/L)	59.97 ± 1.32	60.43 ± 1.26
Creatinine (μmol/L)	66.17 ± 3.37	72.50 ± 2.92
BUN (mmol/L)	4.92 ± 0.25	4.42 ± 0.17
FBG (mmol/L)	5.79 ± 0.36	5.80 ± 0.41
TC (mmol/L)	131.17 ± 3.90	130.03 ± 2.96
TG (mmol/L)	75.03 ± 2.48	79.63 ± 1.72
LDL‐C (mmol/L)	70.00 ± 1.72	72.07 ± 2.66
HDL‐C (mmol/L)	24.77 ± 0.67	25.22 ± 0.61

Note

Data are expressed as mean ± SEM. There were no significant differences in the haematology test indicators between patients in the control group and the nicorandil group.

Abbreviations: ALT, alanine aminotransferase; AST, aspartate aminotransferase; BUN, blood urea nitrogen; FBG, fasting blood glucose; HDL‐C, high‐density lipoprotein cholesterol; LDL‐C, low‐density lipoprotein cholesterol; TC, total cholesterol; TG, total cholesterol; WBC, white blood cell.

Three patients with STEMI in the control group suffered coronary no‐reflow during PCI, and in all three, coronary blood flow returned to TIMI level 3 after intracoronary application of nitroprusside. No patients suffered coronary no‐reflow in the nicorandil group (*P* > .05, vs control).

### 3.2 Effect of nicorandil on the inflammatory response after PCI

In order to observe the effect of nicorandil on PCI‐related inflammation, we tested the expression of pro‐inflammatory factors and anti‐inflammatory factors before PCI, 24 hours after PCI and 7 days after PCI. Pro‐inflammatory cytokines included: TNF‐α, IL‐6, IL‐1β, VCAM‐1, ICAM‐1, CXCL‐1, CXCL‐2, MCP‐1 and IL‐18. Anti‐inflammatory factors included: IL‐10, IL‐19, IL‐33 and IL‐1RA.

**Figure 1:**
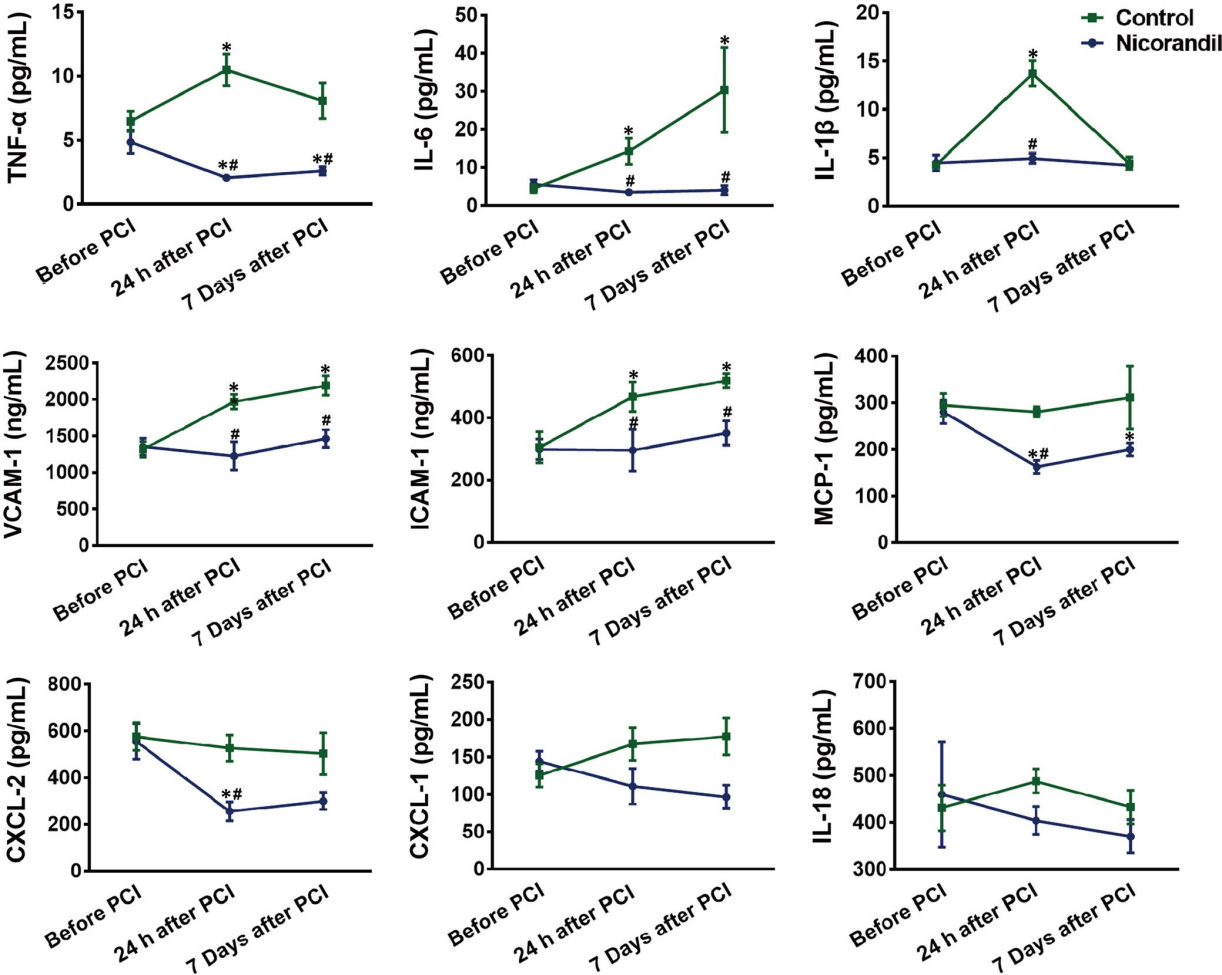
The expression of inflammatory factors of patients before and after PCI. The expression of inflammatory factors including TNF‐α, IL‐6, IL‐1β, VCAM‐1, ICAM‐1, CXCL‐1, CXCL‐2, MCP‐1 and IL‐18 before PCI, 24 hours and 7 days after PCI, in patients undergoing PCI with or without nicorandil application. *P ＜ .05 *vs* before PCI; ^#^ *P *＜ .05 *vs* the control group

We found there were no significant differences in the expression of both pro‐inflammatory and anti‐inflammatory cytokines between the patients in the two groups before PCI. For patients in the control group, the expression of pro‐inflammatory cytokines, including TNF‐α, IL‐6, IL‐1β, VCAM‐1 and ICAM‐1, were remarkedly elevated 24 hours after PCI (*P* < .05, *vs* before PCI, Figure 1). The elevated expression of IL‐6, VCAM‐1 and ICAM‐1 continued until 7 days after PCI (Figure 1). In contrast, patients in the nicorandil group did not show a significant increase in the expression of these inflammatory factors 24 hours after PCI (*P* > .05, *vs* before PCI). The expression of TNF‐α, IL‐6, IL‐1β, VCAM‐1, ICAM‐1, CXCL‐2 and MCP‐1 were much lower than those of the patients in the control group 24 hours after PCI (*P* < .05, Figure 1).

**Figure 2:**
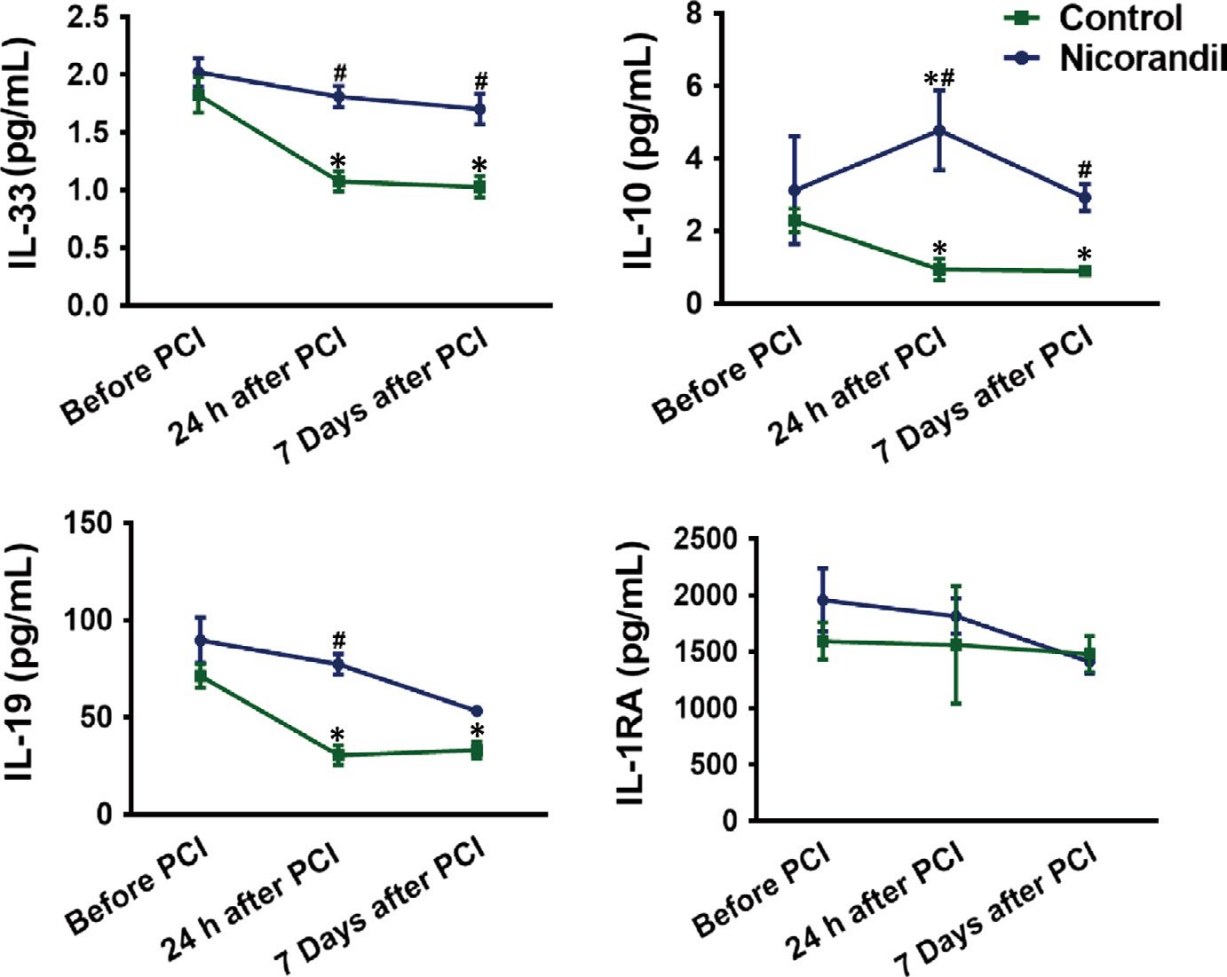
The expression of anti‐inflammatory factors of patients before and after PCI. The expression of anti‐inflammatory factors including IL‐10, IL‐19, IL‐33 and IL‐1RA before PCI, 24 hours and 7 days after PCI, in patients undergoing PCI with or without nicorandil. *P ＜ .05 *vs* before PCI; ^#^ *P *＜ .05 *vs* the control group

For patients in the control group, the expression of the anti‐inflammatory cytokines, including IL‐33, IL‐10 and IL‐19, were remarkedly decreased 24 hours after PCI and continued so until 7 days after PCI (*P* < .05, *vs* before PCI). In contrast, the expression of IL‐33 and IL‐19 of the patients in the nicorandil group did not show a significant decrease 24 hours after PCI, compared with those before PCI (*P* > .05, Figure 2). The expression of IL‐10 24 hours after PCI was much higher than that before PCI (*P* < .05).

These results suggested that nicorandil regulated the inflammatory response induced by PCI, which might be an important mechanism of nicorandil in improving coronary microcirculation, in addition to dilating the coronary microvasculature.

### 3.3 Possible mechanisms of nicorandil in regulating PCI‐induced inflammation

**Figure 3:**
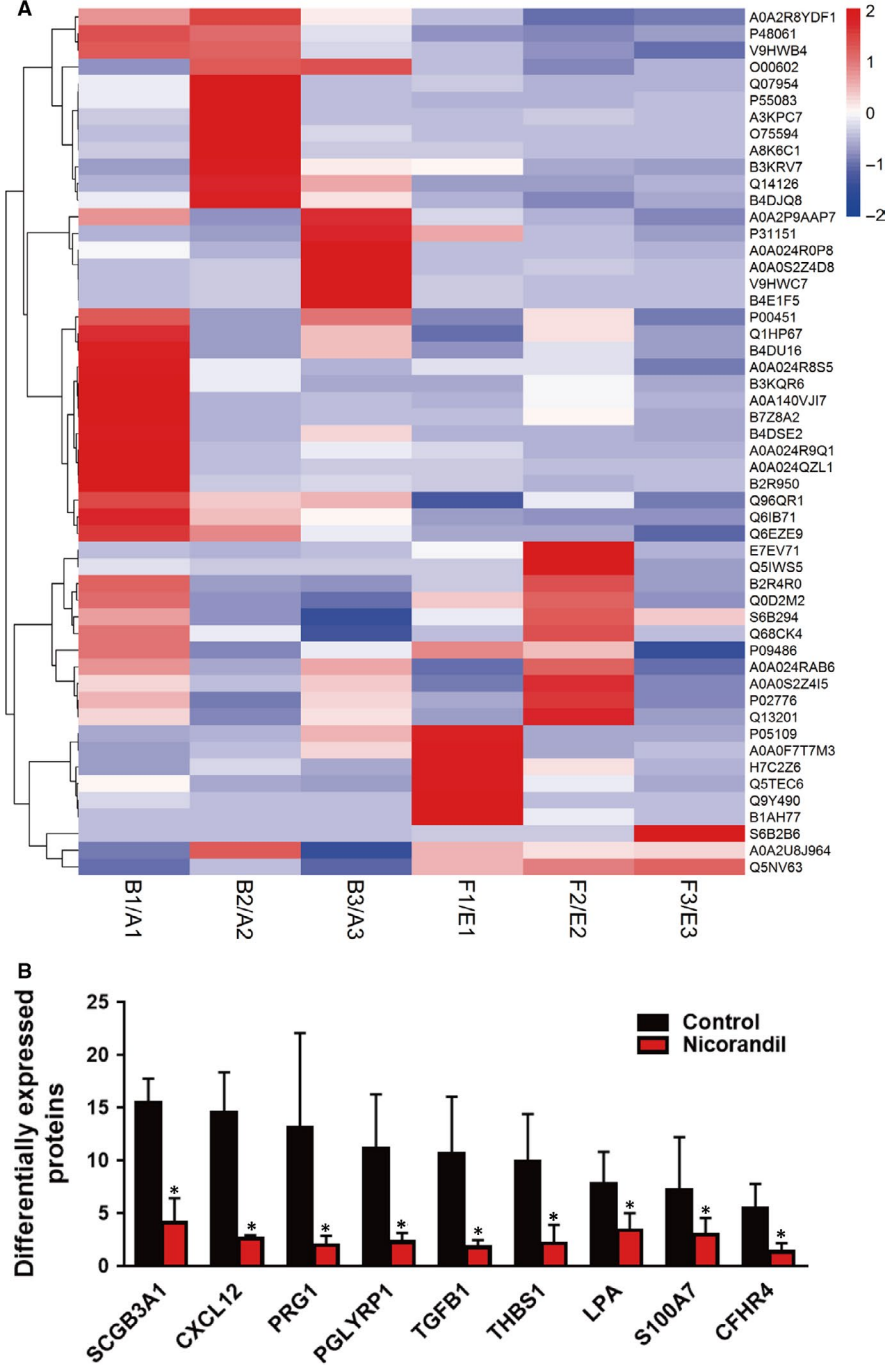
Results of a cluster analysis based on samples and all significantly altered proteins. A, The colour gradient represents the expression levels of genes, as indicated on the right side. Each group was biologically repeated three times. B, Bar chart depiction of proteins that are significantly changed after PCI, and for which nicorandil treatment can partly reverse this effect

To further elucidate the potential mechanism involved in the anti‐inflammatory function of nicorandil, we performed proteomic analysis with the serum collected before, and 24 hours after, PCI in both the control and nicorandil groups. Differentially expressed proteins (DEPs) were defined as those showing a fold change (FC) >2 or <0.5 in relative abundance. In pairwise comparisons, we identified 53 proteins that were significantly changed 24 hours after PCI in the control group, which were reversed in the nicorandil group (Figure 3A). Among these 53 detected proteins, 39 were up‐regulated after PCI in the control group, but down‐regulated in nicorandil group, and 14 proteins were down‐regulated significantly after PCI in control group, and the changes were reversed in the nicorandil group (Figure 3A).

For a more detailed view on proteins for which nicorandil rescues, we screened out those genes that were (a) significantly changed after PCI (ie FC＞5 as compared to control) and (b) for which nicorandil administration distinctly increased or decreased this effect. These criteria were fulfilled by nine proteins (Figure 3B). Further analysis revealed that two proteins were involved in the immune response including secretoglobin 3A1 (SCGB3A1) and peptidoglycan recognition protein 1 (PGLYRP1). Two of these proteins are associated with inflammation, including the CXC chemokine ligand 12 (CXCL12) and transforming growth factor beta 1 (TGF‐β1). Lysophosphatidic acid (LPA) and thrombospondin‐1 (THBS1) are related to platelet activation. PACAP‐responsive gene 1 (PRG1) and S100 calcium‐binding protein A7 (S100A7) are closely associated with malignant tumours. Complement factor H‐related 4 (CFHR4) is a complement‐related protein.

**Figure 4:**
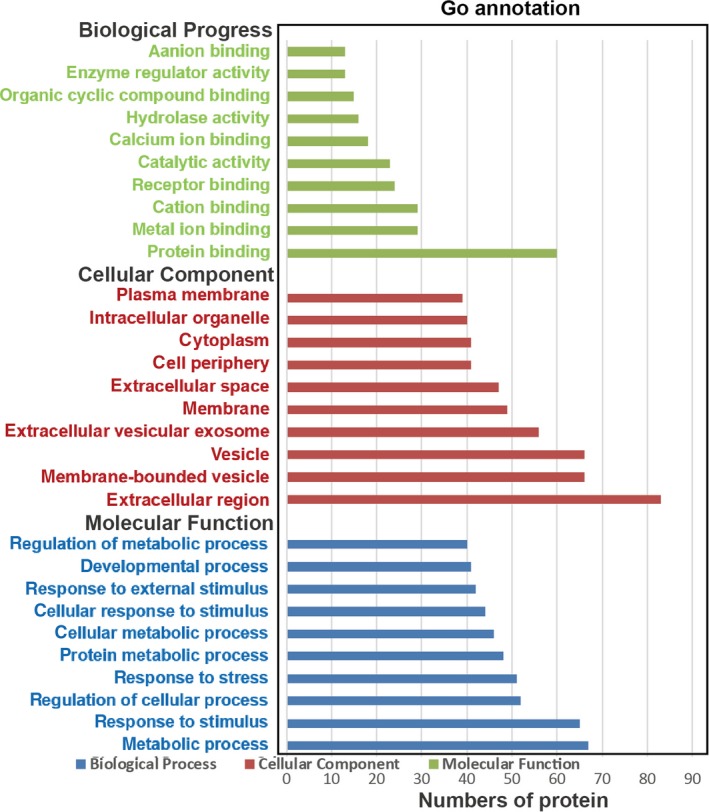
GO analysis of the differentially expressed proteins

**Figure 5:**
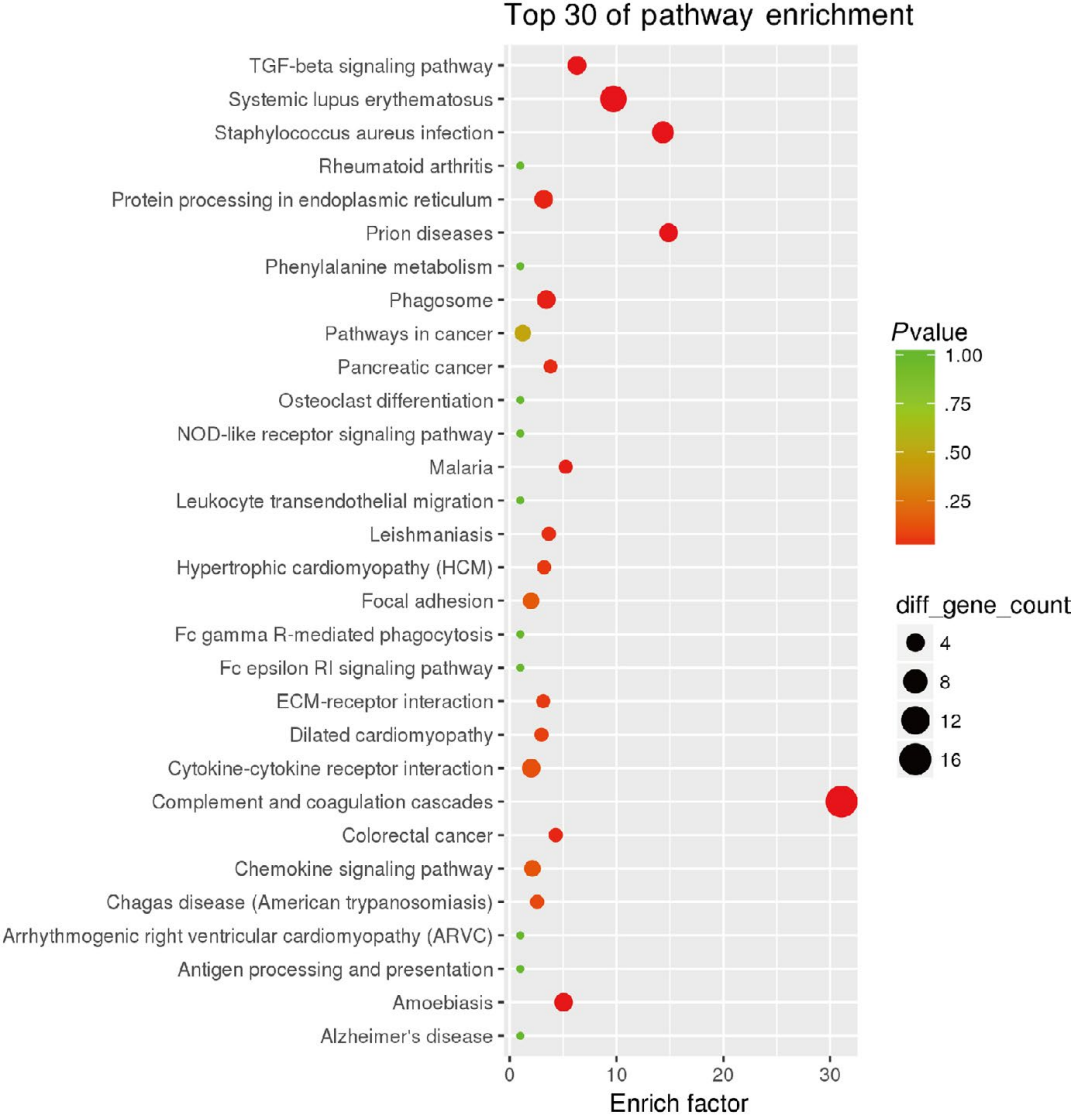
Pathway enrichment analysis. As the enrichment increases, the corresponding function is more specific

The Gene Ontology (GO) analyses indicated that the majority of DEPs had an extracellular distribution (Figure 4). The Kyoto Encyclopedia of Genes and Genomes (KEGG) analyses demonstrated that the DEPs were significantly enriched in the complement and coagulation cascades, infection and TGF‐beta signalling pathway (Figure 5).

## 4 DISCUSSION

This study aimed to explore the potential mechanism(s) of nicorandil in preventing MNR, and in dilating coronary microvasculature. We found that intracoronary application of nicorandil in ACS patients before PCI could significantly decrease the expression of those pro‐inflammatory cytokines, which were elevated after PCI in patients without nicorandil treatment. We also found that the expression of anti‐inflammatory cytokines, which were decreased after PCI, was enhanced in patients injected with nicorandil. We further performed proteomic analysis to elucidate the potential mechanism(s) involved in the anti‐inflammatory effects of nicorandil and found that the immune response, inflammation and platelet activation might all be involved. We concluded that nicorandil might improve coronary microcirculation and prevent MNR by regulating inflammatory responses after PCI, in addition to dilating coronary microvasculature.

Current research indicates that the inflammatory‐oxidative stress mechanism plays a key role in the development of MNR.^11^, ^12^ During myocardial ischaemia, the function of the oxygen free radical scavenging system is weakened or even disappears altogether. After reperfusion, large amounts of reactive oxygen species (ROS) are generated through multiple channels, overwhelming the antioxidant defence system's ability to scavenge, and causing a large accumulation of ROS.^13^ Mitochondria are at the core of oxidative stress, and the mitochondrial respiratory chain is the main source of ROS, accounting for more than 90% of ROS sources. During ischaemia and hypoxia, due to damage to the mitochondrial respiratory chain, the normal oxidative phosphorylation process weakens, electron leakage from the respiratory chain increases and reaction with molecular oxygen readily occurs, generating oxygen free radicals. During the ischaemia‐reperfusion process, ROS are produced in great quantities through multiple pathways. The endogenous clearance system is overwhelmed, which causes the accumulation of reactive oxygen species in the body and causes oxidative damage.^13^

ATP‐sensitive potassium channel (K_ATP_) complexes are widely distributed in various tissues in the body, including the heart, skeletal muscle, pancreas and arterial smooth muscle. Within the cell, K_ATP_ is found on the cell membrane (sarcolemmal K_ATP_ channel, sK_ATP_) and on the mitochondrial membrane (mitochondrial K_ATP_, mitoK_ATP_). Oxygen free radicals are located downstream of mitoK_ATP,_ 14, 15, 16 and mitoK_ATP_ is directly involved in regulating the production of oxygen free radicals. The opening of mitoK_ATP_ channels can reduce the Ca2 + overload in mitochondria, regulate the generation of oxygen free radicals, reduce swelling of the mitochondrial matrix, optimize the respiratory chain and inhibit apoptosis.^7^ In this sense, the opening of mitoK_ATP_ channels may reduce the ischaemia‐reperfusion myocardial injury by regulating the oxidative stress response and improving the phenomenon of no‐reflow after PCI.

Nicorandil has the dual effects of being nitrate‐like and an ATP‐sensitive potassium channel opener. K_ATP_ is abundantly expressed in small arterial smooth muscle cell membranes with a diameter of about 100 µm. Therefore, nicorandil can expand coronary microcirculation by opening the cell membrane K_ATP_ channels. By opening myocardial mitoK_ATP_ channels, nicorandil also plays a key pharmacological role in ‘ischaemic pre‐conditioning’.^17^ Studies have shown that nicorandil can reduce endothelial function damage caused by coronary stent coating drugs paclitaxel and sirolimus. This endothelial protection is mainly mediated by the opening of mitoK_ATP_ (and not cell membrane KATP).^18^, ^19^ It is speculated that mitoK_ATP_ may exert vascular endothelial cell protection by inhibiting oxidative stress in vivo. Our results showed that the intracoronary administration of nicorandil before PCI can significantly inhibit the expression of pro‐inflammatory factors and enhance the anti‐inflammatory factors after PCI. This suggests that regulation of inflammation might be an important mechanism behind nicorandil's ability to improve coronary microcirculation and prevent coronary no‐reflow.

In summary, in addition to dilating coronary microvasculature, the regulation of inflammatory responses after PCI might be another important mechanism of nicorandil in improving coronary microcirculation after PCI.

## CONFLICT OF INTEREST

The authors confirm that there are no conflicts of interest.

## AUTHOR CONTRIBUTIONS

Su Guohai and An Guipeng designed the research study; Hu Keqing, Wang Xiaoqi and Hu Hongyan screened patients for enrollment and conducted the studies; Xu Zhongyang and Zhang Jiaxing performed the data analysis; and Hu Keqing, An Guipeng and Su Guohai wrote the paper.

## Data Availability

The datasets used and analysed in the current study are available from the corresponding author on reasonable request.
